# Inventory data on Brazilian Amazon׳s non-wood native biomass sources for bioenergy production

**DOI:** 10.1016/j.dib.2018.09.050

**Published:** 2018-09-22

**Authors:** Josmar Almeida Flores, Odorico Konrad, Cíntia Rosina Flores, Nádia Teresinha Schroder

**Affiliations:** a Graduate Program in Environment and Development, Universidade do Vale do Taquari, Lajeado, Rio Grande do Sul, Brazil; b Universidade Federal de Rondônia, Porto Velho, Rondônia, Brazil; c Graduate Program in Health Promotion, Human Development and Society, Universidade Luterana do Brasil, Canoas, Rio Grande do Sul, Brazil

**Keywords:** Brazilian Amazon, Bioenergy, Non-wood biomass, Protected areas, Renewable sources

## Abstract

This data article presents a set of non-wood native biomass sources identified for bioenergy production in isolated communities living in Amazon׳s extractive reserves. The data were inventoried using management plan documentation, which provides technical information on Amazon׳s protected areas. The sample was collected from a virtual database published by the Ministry of Environment, the federal body responsible for managing protected areas in Brazil. Five variables were extracted from the management plans to produce the inventory, which includes data on biomass typology, nomenclature and occurrence, as well as mode of access to communities and availability of energy sources in the protected areas.

**Specifications table**

**Table:** 

Subject area	*Renewable energy, environment*
More specific subject area	*Native biomass for bioenergy production*
Type of data	*Tables, text file*
How data was acquired	*Management plan documentation was downloaded from a virtual database published by the Brazilian Ministry of Environment*
Data format	*Raw data were processed and filtered*
Experimental factors	*Variables regarding the protected areas were selected from management plan documentation*
Experimental features	*Data are focused on determining Amazon׳s non-wood native biomass sources to be used for bioenergy production in isolated communities living in extractive reserves*
Data source location	*Brazilian Amazon*
Data accessibility	*Data are available in this article*
Related research article	*None*

**Value of the data**

• The present data provide information on non-wood native biomass sources that are locally available in extractive reserves of a developing country.
• The data support the use of locally available non-wood biomass materials as a renewable source for bioenergy generation.
• The data can be used for comparison with other non-wood native biomass sources.
• The data may contribute to reducing the use of fossil fuels in Amazon׳s protected areas.

## 1 Data

This data article consists of an inventory of Amazon׳s non-wood native biomass sources for bioenergy production in isolated communities living in extractive reserves. The data indicate the types of non-wood native biomass sources identified through analysis of management plan documentation of the Brazilian Amazon׳s protected areas, including popular and scientific names as well as their measured occurrence in extractive reserves (Table 1).

**Table 1:** Non-wood native biomass resources in the Brazilian Amazon.

Scientific name	Popular name	Occurrence in extractive reserves
Alibertia edulis	Apuruí	2
Anacardium giganteum W. Hancock ex Engl.	Caju-açu	1
Anacardium spruceanum Benth. ex Engl.	Cajuí	5
Astrocaryum aculeatum G. F. W. Meyer.	Tucumã	12
Astrocaryum gynacanthum Mart.	Mumbaca	1
Astrocaryum jauari Mart.	Jauari	5
Attalea butyracea	Jaci	4
Attalea paripa	Inajá	4
Attalea phalerata Mart. ex Sprengel	Urucuri	7
Attalea speciose	Babaçu	3
Bactris gasepaes H. B. K.	Pupunha	10
Bactris monticola Barb. Rod.	Marajá	5
Bertholletia excelsa H. B. K.	Castanheira	13
Bixa orellana L.	Urucum	2
Brosimum uleanum	Manitê	1
Byrsonima crassifolia L.	Muruci	2
Caryocar glabrum (Aubl.) Pers. Subsp glabrum	Piquiarana	2
Caryocar villosum (Aubl.) Pers.	Piqui/Piquiá	8
Caryodendron sp.	Castainha	1
Castilla ulei Warburg.	Caucho	1
Chrysobalanus icaco L.	Ajirú	1
Couepia bracteosa Benth	Pajurá	1
Coumo utilis Muell. Arg	Sorva	1
Couroupita guianensis Aubl.	Coité de macaco	1
Dipteryx odorata (Aubl.) Willd	Cumaru Ferro	3
Eccilnusa sp.	Cagaça	1
Elaeis oleifera	Caiaué	1
Eschweilera odorata (Poepp.) Miers	Matamatá	3
Eugenia sp.	Araçá	1
Euterpe oleracea	Açaí	3
Euterpe precatoria M.	Açaí	10
Euterpe sp.	Açaí	4
Faramea sp.	Gurdião	1
Ficus sp.	Gameleira	1
Garcinia madruno (Kunth) Hammel	Bacurizinho	1
Genipa americana L.	Jenipapo	3
Guateria sp.	Araticum	1
Hirtella sp.	Macucu	1
Hymenaea sp.	Jatobá	12
Inga sp.	Ingá	6
Inga velutina	Ingá peluda	1
Jacaratia spinose	Jaracatiá	1
Lecythis Pisonis	Castanha Sapucaia	1
Matisia cf. cordata Humb. & Bonpl.	Sapota	1
Mauritia flexuosa L.f.	Buriti	13
Maximiliana maripa	Inajá	1
Minquartia guianensis Aubl.	Acariquara	4
Oenocarpus bacaba M.	Abacaba/Bacaba	13
Oenocarpus bataua Mart.	Patauá	12
Oenocarpus minor Mart.	Bacabinha	2
Oenocarpus sp.	Bacabão	1
Onychopetalum lucidum R. E. Fries	Envira-caju	2
Orbignya phalerata	Babaçu	11
Orbignya speciose	Babaçu	2
Pachira aquatica Aubl.	Manorana	1
Phytelephas macrocarpa	Jarina	1
Platonia insignis Mart.	Bacuri	7
Pourouma cecropiifolia Mart.	Mapati	2
Pouteria bilocularis	Biorama	1
Pouteria caimito (Ruiz & Pav.) Radlk	Abiu	2
Pseudomedia sp.	Pama	4
Rollinia exsucca (Dun.) DC.	Ata	1
Rollinia mucosa (Jacq.) Baill.	Biribá	1
Scheelea martina	Palheira Uricuri	1
Spondia sp.	Cajá	5
Spondia testudines	Cajarana	1
Spondias mombin L.	Tapereba	5
Stryphnodendron sp.	Bajinha	1
Theobroma cacao L.	Cacau	9
Theobroma grandiflorum (Willd. ex Spreng.) Schum.	Cupuaçu	8
Theobroma microcarpum M.	Cacaurana	1
Theobroma subincanum Mart.	Cupui	1
Theobroma sylvestris Mart.	Cacaui	1
Trema micrantha	Piriquiteira	1
Zollernia paraensis	Jenipapinho	1

Total number of non-wood native biomass resources ……………………………………………………………………………… 75		

Table 2 shows the modes of access to conservation units and the availability of energy sources for the isolated communities living in the extractive reserves. If energy is available, it also includes which energy source is used (fossil, renewable or public distribution network) and who bears the cost (public if provided by the state or private if shared by local residents).

**Table 2:** Extractive reserves and number of non-wood native biomass resources.

Extractive reserve	No. of biomass resources	Mode of access	Energy		
			Supply	Source	Cost
Arapixi	31	Fluvial	Deficient	Network/fossil	Public
					Private
Auatí-Paraná	14	Fluvial	Deficient	Fossil	Private
Baixo Juruá	19	Fluvial	Deficient	Mostly fossil	Private
Barreiro das Antas	12	Fluvial	Nonexistent	–	–
Cazumbá-Iracema	28	Terrestrial and fluvial	Deficient	Fossil	Private
Chico Mendes	15	Terrestrial and fluvial	Deficient	Network/fossil	Public
					Private
Lago do Capanã Grande	23	Fluvial	Deficient	Fossil	Private
Marinha de Caeté-Taperaçu	14	Terrestrial and fluvial	Deficient	Network	Public
Médio Juruá	10	Fluvial	Deficient	Fossil	Private
Rio Gregório	21	Fluvial	Deficient	Fossil	Private
Rio Iriri	10	Fluvial and aerial	Deficient	Fossil	Private
Rio Jutaí	10	Fluvial	Deficient	Fossil	Private
Rio Ouro Preto	15	Terrestrial and fluvial	Deficient	Mostly fossil	Private
Rio Unini	6	Fluvial and aerial	Deficient	Fossil	Private
Rio Xingu	12	Fluvial	Deficient	Fossil	Private
Riozinho do Anfrisio	9	Fluvial, aerial, and Terrestrial	Nonexistent	–	–
Tapajós-Arapiuns	13	Fluvial	Deficient	Fossil	Private

Management plan data were collected, processed and shared in the supplementary material of this data article. The data set consists of a Microsoft Excel spreadsheet (XLSX file) containing raw extracted data, and a detailed description presented in Table 1, Table 2 of this article.

## 2 Experimental design, materials, and methods

This article includes qualitative and quantitative data collected from technical documents, or management plans, of Amazon׳s extractive reserves. Of the 50 extractive reserves in the Brazilian Amazon, 33 have not published the referred document (Table 3); thus, the present sample consists of 17 management plans (Table 4).

**Table 3:** Amazon׳s extractive reserves without a management plan.

Extractive reserve	Covered municipality(ies)	State	Created on	No. of hectares
Alto Juruá	Marechal Thaumaturgo Jordão/Porto Walter/Tarauacá	AC	1/23/1990	537,949.36
Alto Tarauacá	Tarauacá/Jordão/Marechal Thaumaturgo	AC	11/8/2000	150,924.09
				
Riozinho da Liberdade	Cruzeiro do Sul/Tarauacá/Marechal Thaumaturgo/Porto Walter	AC	2/17/2005	324,905.93
		AM		
Canutama	Canutama/Lábrea/Tapauá	AM	3/27/2009	197,987
Catuá-Ipixuna	Coari/Tefé	AM	9/5/2003	217,486
Guariba	Apuí/Manicoré	AM	6/1/2005	150,465
Ituxí	Lábrea	AM	6/5/2008	776,329.64
Médio Purús	Pauini/Lábrea/Tapauá	AM	5/8/2008	604,235.97
Rio Cajari	Mazagão/Laranjal do Jari/Vitória do Jari	AP	3/12/1990	532,404.51
Ciriaco	Cidelândia/Imperatriz	MA	6/17/2010	8106.63
Cururupu	Apicum-Açu/Bacuri/Serrano do Maranhão/Cururupu/Porto Rico do Maranhão	MA	6/2/2004	186,056.73
Quilombo Flexal	Mirinzal	MA	5/20/1992	9338.45
Guariba-Roosevelt	Aripuanã/Colniza/Rodolândia	MT	6/19/1996	138,092
Arióca Pruanã	Oeiras do Pará/Bagre	PA	11/16/2005	83,816.72
Chocoaré-Mato Grosso	Santarém Novo/São João das Pirabas/Maracanã/Igarapé-Açú	PA	12/16/2002	2783.20
Gurupá-Melgaço	Melgaço/Gurupá/Breves	PA	11/30/2006	145,574.11
Ipaú-Anilzinho	Baião	PA	6/14/2005	55,835.01
Mãe Grande de Curuçá	Curuçá/Marapanim/São Caetano de Odivelas/São João da Ponta	PA	12/13/2002	36,678.78
Mapuá	Breves Anajás	PA	5/20/2005	93,747.66
Maracanã	Maracanã/Igarapé-Açú/Magalhães Barata/Salinópolis/Santarém Novo/São João Pirabas	PA	12/13/2002	30,179.65
Marinha Cuinarana	Magalhães Barata	PA	10/10/2014	11,036.41
Marinha de Araí-Peroba	Augusto Corrêa	PA	5/20/2005	62,578.12
Marinha de Gurupi-Piriá	Viseu/Augusto Correia	PA	5/20/2005	72,789.93
Marinha de Soure	Soure	PA	11/22/2001	29,578.80
Marinha de Tracuateua	Tracuateua/Bragança/Quatipuru	PA	5/20/2005	27,864.50
Marinha Mestre Lucindo	Marapanim	PA	10/10/2014	26,464.88
Marinha Mocapajuba	São Caetano de Odivelas	PA	10/10/2014	21,027.80
Renascer	Prainha	PA	6/5/2009	209,667.37
São João da Ponta	São João da Ponta/São Caetano de Odivelas/Curuçá	PA	12/13/2002	3409.49
Terra Grande Pracuúba	São Sebastião da Boa Vista Curralinho/Anajás/Breves/Muaná	PA	6/5/2006	194,870.38
Verde para Sempre	Porto de Moz/Gurupá/Brasil Novo/Prainha	PA	11/8/2004	1,289,000.00
Lago do Cuniã	Porto Velho	RO	11/10/1999	50,604.27
Rio do Cautário	Guajará-Mirim/Costa Marques	RO	8/7/2001	75,125.50

**Table 4:** Amazon׳s extractive reserves with a management plan.

Extractive reserve	Covered municipality(ies)	State	Created on	No. of hectares
Cazumbá-Iracema	Manoel Urbano/Sena Madureira	AC	10/19/2002	754,987.10
Chico Mendes	Sena Madureira/Rio Branco/Capixaba/Xapuri/Brasiléia/Assis Brasil/Epitaciolândia	AC	3/12/1990	931,542.94
Arapixi	Boca do Acre	AM	6/21/2006	133,711.51
Auatí-Paraná	Japurá/Fonte Boa/Maraã	AM	8/7/2001	146,949.38
Baixo Juruá	Juruá/Uarini	AM	8/1/2001	178,038.92
Lago do Capanã Grande	Manicoré/Tapauá/Beruri	AM	6/3/2004	304,313.44
Médio Juruá	Carauari	AM	3/4/1997	286,954.81
Rio Gregório	Eirunepé	AM	4/25/2007	427,004
Rio Jutaí	Jutaí	AM	7/16/2002	275,515.82
Rio Unini	Barcelos/Maraã	AM	6/21/2006	849,693.35
Marinha de Caeté-Taperaçu	Bragança/Tracateua	PA	5/20/2005	42,489.81
Rio Iriri	Altamira	PA	6/5/2009	398,997.61
Rio Xingu	Altamira/São Félix do Xingú	PA	6/5/2008	303,004.70
Riozinho do Anfrisio	Altamira/Itaituba/Rurópolis/Trairão	PA	11/8/2004	736,144.01
Tapajós-Arapiuns	Santarém/Aveiro	PA	11/6/1998	677,521.47
Barreiro das Antas	Guajará-Mirim	RO	8/7/2001	106,198.52
Rio Ouro Preto	Guajará-Mirim/Nova Mamoré	RO	3/13/1990	204,632.81

Thirty out of the 33 conservation units without a management plan are not complying with the 5-year deadline to prepare the document, according to current legislation [1]. The remaining extractive reserves, namely Marinha Cuinarana (state of Pará, PA), Marinha Mestre Lucindo (PA), and Marinha Mocapajuba (PA), are within the established deadline, as they were created in 2014.

The management plans of the conservation units were collected from a virtual database published by the Brazilian Ministry of Environment, the federal body responsible for managing protected areas in Brazil. Data collection was based on the inventory of the following variables: (i) non-wood native biomass resources, including popular and scientific names of species; (ii) type of access to the conservation unit; (iii) energy availability in the isolated communities living in extractive reserves; and, in case it is available, (iv) which energy source is used (fossil, renewable, or public distribution network) and (v) who bears the cost (public sector or private households).
